# Danshensu methyl ester enhances autophagy to attenuate pulmonary fibrosis by targeting lncIAPF–HuR complex

**DOI:** 10.3389/fphar.2022.1013098

**Published:** 2022-10-31

**Authors:** Qi Zhu, Jing Wang, Yunxia Ji, Jianlin Luan, Dayong Yue, Weili Liu, Hongbo Li, Jinjin Zhang, Guiwu Qu, Changjun Lv, Xiaodong Song

**Affiliations:** ^1^ Department of Cellular and Genetic Medicine, School of Pharmaceutical Sciences, Binzhou Medical University, Yantai, China; ^2^ Department of Respiratory and Critical Care Medicine, Binzhou Medical University Hospital, Binzhou Medical University, Binzhou, China; ^3^ Medical Research Center, Binzhou Medical University, Yantai, China; ^4^ School of Gerontology, Binzhou Medical University, Yantai, China

**Keywords:** pulmonary fibrosis, danshensu, lncRNA, autophagy, HuR (ELAVL1)

## Abstract

Pulmonary fibrosis is an irreversible fibrotic process that has a high mortality rate and limited treatment options; thus, developing a novel therapeutic drug is critical. In this study, we synthesized danshensu methyl ester (DME) and explored its anti-pulmonary fibrotic ability on TGF-β1-stimulated lung fibroblast *in vitro* and on bleomycin-induced pulmonary fibrosis *in vivo*. Results showed that DME decreased the expression of differentiation-related proteins, including fibroblast activation protein 1 (FAP1) and S100 calcium-binding protein A4 (S100A4), and fibrotic markers, such as *a*-SMA, vimentin, and collagen *in vivo* and *in vitro*. In addition, DME markedly repressed myofibroblast proliferation and migration. Mechanistically, chromatin immunoprecipitation–PCR, RNA immunoprecipitation, half-life, and other experiments revealed that DME inhibited activating transcription factor 3 expression *via* TGF-β1 signal transduction leading to a decrease in lncIAPF transcription and stability. Moreover, DME blocked human antigen R (HuR) nucleocytoplasmic translocation and promoted its degradation *via* downregulating lncIAPF, which markedly decreased the expression of HuR target genes such as negative autophagic regulators (EZH2, STAT1, and FOXK1). Collectively, our results demonstrated that DME enhanced autophagy to attenuate pulmonary fibrosis *via* downregulating the lncIAPF–HuR-mediated autophagic axis and the lncIAPF–HuR complex can be the target for drug action.

## Introduction

Pulmonary fibrosis is the common end stage for many diseases, such as Sjogren’s syndrome, systemic lupus erythematosus, dermatomyositis, and rheumatoid arthritis. It is primarily characterized by morphological and functional abnormalities within the lung. Morphological abnormalities include progressive deposition of the extracellular matrix (ECM), thickening of the alveolar wall, and a large number of fibroblast proliferation and migration. Functional abnormalities include the decline in forced vital capacity (FVC), vital capacity, total lung capacity, and diffusion capacity of the lungs for carbon monoxide (Johannson et al., 2021). Environmental exposure, genetic factors, and aging have been described as potential risk factors (Moss et al., 2022). In addition, acute respiratory syndrome coronavirus can cause pulmonary fibrosis in many patients (Aveyard et al., 2021; Bando et al., 2022). Despite major advances in the mechanism of pulmonary fibrosis, no effective therapeutic methods have been developed. Therefore, research on effective treatment is of great practical importance.

At present, long non-coding RNAs (lncRNAs) are considered as RNA transcript with >200 nucleotides, which does not encode protein. They are multifaceted, versatile regulators of most cellular processes, including cell death, proliferation, migration, and differentiation. Accumulating studies have revealed that lncRNAs contribute to disease initiation, progression, and metastasis by modulating transcription, translation, posttranslational modification, and epigenetic modification and to protein/RNA stability by interacting with DNA, RNA, and/or proteins (de Goede et al., 2021; Quinodoz et al., 2021). For example, Hua et al. have revealed that a risk SNP-mediated promoter–enhancer switching can promote the initiation and progression of aggressive prostate cancer through lncRNA-PCAT19 (Hua et al., 2018). lncRNA-EPS serves as a transcriptional brake to restrain inflammation (Atianand et al., 2016). Similarly, lncRNA application in pharmaceutical research can be a powerful tool for drug research, such as the validation of drug targets and the study of resistance mechanisms and drug toxicity. For example, an integrated genome-wide CRISPRa approach is developed to functionalize lncRNAs in drug resistance (Bester et al., 2018). Chemotherapy-induced lncRNA-CILA1 promotes chemo-resistance in tongue squamous cell carcinoma (TSCC), and it is a therapeutic target for TSCC treatment (Lin et al., 2018). In addition, lncRNA-HIF1A-AS2 induces osimertinib resistance in patients with lung adenocarcinoma by regulating the miR-146b-5p/IL-6/STAT3 axis (Si et al., 2021). Therefore, understanding lncRNA-mediated mechanisms of drug responses will improve responses to chemotherapy and outcomes of disease treatment. In pulmonary fibrosis, RNA-binding motif protein 7 promotes the development of fibrosis by controlling the expression of selected target lncRNA-NEAT1 (Fukushima et al., 2020). lncRNA-DNM3OS promotes pulmonary fibrogenesis in trans by producing three miRNAs (i.e., miR-199a-5p/3p and miR-214-3p). Pharmacological approaches aiming at interfering with DNM3OS not only prevent lung fibrosis but also improve established pulmonary (Savary et al., 2019). To date, many lncRNA-based therapies are being investigated, including gene therapy, medical treatment, and stem cell therapy.

**FIGURE 1:**
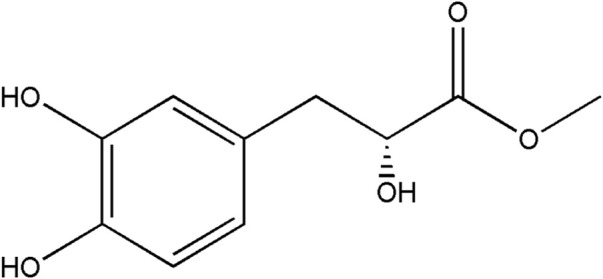
Molecular formula of DME (C10H12O5).

lncIAPF, as a pro-fibrotic factor, can be presented in both nucleus and cytoplasm in normal lung tissue. Human antigen R (HuR), as an RNA binding protein, enhances the stability of RNA and increases its expression. In a recent study (Zhang Q et al., 2022), lncIAPF and HuR function as lncIAPF-HuR complex to accelerate pulmonary fibrosis by blocking autophagy. But, whether lncIAPF-HuR complex can be a drug target is yet to be explored. Our team once has extracted danshensu from salvia miltiorrhiza, which has the effect of anti-pulmonary fibrosis. But it also exhibits physical and chemical defects, such as poor chemical stability, easy oxidation, and discoloration, which limit its clinical application (Zhao et al., 2018). Then we prepared a new compound based on danshensu (Figure 1). Because it is an esterified derivative of danshensu, we named it danshensu methyl ester (DME). In the present study, we further investigated the anti-pulmonary fibrosis and mechanism of DME targeting lncIAPF–HuR complex in TGFβ1-stimulated lung fibroblast and bleomycin (BLM)-treated mice.

## Materials and methods

### Animal model and ethical statement

Animal experiments were carried out in accordance with the regulation of the Animal Experiment Ethics Committee of Binzhou Medical College. 8-week-old C57/BL6 mice with an average weight of 20 ± 5 g were divided into 5 groups: sham group, bleomycin (BLM) group, BLM + danshensu methyl ester (DME) treatment group (5, 10, 20 mg/kg). The BLM model group was sprayed with 5 mg/kg BLM through the trachea by using Penn-Century MicroSprayer (Penn-Century Inc., Wyndmoor, PA, United States). The sham group was sprayed with the same amount of normal saline. The DME group was treated with different concentrations DME by intraperitoneal injection after BLM spraying. After 28 days of modeling, the lung tissue of the right lobe of mice was frozen in liquid nitrogen to extract tissue protein and RNA. The lung tissue of the left lobe was perfused and fixed with 4% paraformaldehyde for follow-up experiments.

### Cell model

Human fetal lung fibroblast MRC-5 cells were purchased from the American Type Culture Collection and cultured in advanced minimum essential medium containing 10% fetal bovine serum, 1% GlutaMAX, 1% sodium pyruvate, 1% NEAA, and 100 × penicillin/streptomycin solutions at 37°C and 5% CO_2_ incubator. The cells were divided into different groups according to the experimental requirement: normal group, TGF-β1 treatment group, TGF-β1 + DME treatment group. Cells were treated with or without 5 ng/ml TGF-β1 for 72 h, and then treated with or without DME for 48 h. For rescue assay, cells were treated with TGF- β1 for 72 h and then transfected with an overexpression lncIAPF plasmid for 6 h, followed by treatment with DME for 48 h.

### Cell counting kit-8 (CCK-8) toxicity experiment

5 × 10^3^ MRC-5 cells were cultured for 24 h in the 96-well plates. 100 μl different concentrations of DME was added to each well and cultured for 24 h or 48 h. Then 10 μl CCK-8 solution was added to each well. After 4 h, the absorbance was measured at 450 nm with a microplate reader. Cell viability was calculated according to the kit formula.

### Wound healing assay

MRC-5 cells were seeded in 96-well plates. When the cell density reached 60%, the complete medium was replaced by serum-free medium with or without TGF-β1 for 72 h. After an even line was drew on the surface of cultured cells, cells were washed three times with 1 × PBS to wash away the dead cells. Then, the different concentrations of DME were added to the plates and the plates were incubated in an IncuCyte S3 live-cell analysis system (Essen BioScience). The instrument automatically monitored and analyzed the live cells growth.

### Hematoxylin-eosin staining and Masson’s trichrome staining

The fixed lung tissue was dehydrated, soaked in paraffin overnight, embedded in paraffin, cut into 4 μM sections with a Leica microtome (RM2255), and stained with H&E or Masson staining kits (Solarbio, China), respectively. The paraffin sections were dewaxed and stained, dehydrated with absolute ethanol, transparent in xylene, dripped with neutral gum, and sealed with a cover glass. The lung tissues of each group were observed under a light microscope.

### Quantitative real-time reverse transcription PCR assay

Total RNA was extracted from MRC-5 cells or lung tissues with Trizol, then reverse transcribed into cDNA by reverse transcription kit (TaKaRa Biotechnology). A total volume of 20 μL system was as the following: 2 μl cDNA, 7.2 μl RNA free water, 0.4 μl lncIAPF/GAPDH forward primer, 0.4 μl lncIAPF/GAPDH reverse primer, 10 μL SYBR^®^ Premix Ex TaqTM. The reaction program was as the following: holding temperature at 95°C for 30 s. 45 cycles of PCR amplification at 95°C for 5 s, 60°C for 20 s and 72°C for 30 s.

### Immunofluorescence observation

MRC-5 cells were seeded on the cell slides in a 24-well plate, fixed with 4% tissue cell fixative for 30 min, and punched with 0.3% TritonX-100. The slides were washed with 1 × PBS, blocked with goat serum for 1 h, and incubated with anti-HuR (1:1,000, Proteintech, China) at 4°C overnight. Fluorescently labeled secondary antibody was added, incubated at room temperature for 60 min in the dark, and then discarded. 200 μl DAPI was added to each well to stain cell nuclei for 6 min and washed with 1 × PBS. Finally, the anti-fluorescence quencher was dropped on the cell slides. All images were collected under a laser scanning confocal microscope (Zeiss LSM880, Germany).

### Western blot

Cells or lung tissues were harvested and lysed in radio immunoprecipitation assay buffer and phenylmethanesulfonyl fluoride (100:1). The protein concentration was measured using bicinchoninic acid protein assay kit (Coolaber, China). After separation in sodium dodecyl sulfate-polyacrylamide gel electrophoresis, the proteins were transferred to a polyvinylidene fluoride membrane, and the protein bands were blocked with 5% nonfat dry milk for 2 h, and mixed with anti-collagen III (1:1,000, Affinity, China), anti-collagen I (1:1,000, Affinity, China), anti-α-SMA (1:1,000, Affinity, China), anti-Vimentin (1:1,000, Affinity, China), Anti-GAPDH (1:10,000, Affinity, China), anti-ULK1 (1:1,000, Affinity, China), anti-FAP1 (1:1,000, Cell Signaling, United States), anti-TGF-β1, anti-ATG5, anti-DRAM2 (1:1,000, Cell Signaling, United States) 1,000, Bioss, China), anti-GABARAP (1:1,000, Affinity, China), anti-EZH2, anti-FOXK1, anti-STAT1 (1:1,000, Abcam, United Kingdom), anti-P62, anti-HuR (1:1,000, Proteintech, China)), anti-ATF3 (1:1,000, Proteintech, China), anti-LC3 polyclonal antibody and incubated overnight. Membranes were washed three times with 1× tris buffered saline tween and then incubated with goat anti-rabbit/mouse secondary antibody for 1 h. Finally, protein expression was detected by enhanced chemiluminescence kit (Spark Jade, China).

### Nuclear and cytoplasmic extraction

The cell samples collected in 1.5 ml centrifuge tubes. Pre-cooled cytoplasmic extraction reagent (CER) I and CER II were added and mixed. After centrifugation, the supernatant was aspirated to obtain the cytoplasmic extraction. Then pre-cooled nuclear extraction reagent was added to the remaining precipitation, mixed and incubate on ice for 10 min. After centrifugation, the supernatant was aspirated to obtain the nucleus extraction.

### Dual fluorescence HBAD-mcherry-EGFP-LC3 detection

MRC-5 cells were seeded in glass-bottom dishes. When the cell density reached 60%–70%, the complete medium was replaced with 500 μl serum-free medium. 5 μl HBAD-mcherry-EGFP-LC3 (HANBIO, China) was added into the medium. After 6 h, the medium containing HBAD-mcherry-EGFP-LC3 was removed. Then the cells were treated with/without TGF-β1 and medium containing DME for 48 h. Fluorescence images were examined under a laser scanning confocal microscope.

### Half-life analysis

1 × 10^6^/ml MRC-5 cells were seeded in cell dishes. Cells were treated with TGF-β1 for 72 h, and then treated with DME for 48 h. Finally, 2 ml serum-free medium containing 5 μg/ml actinomycin D was added into the cell samples for different times (0, 1, 2, 3, 4 h). Cells were collected and total RNA was extracted for subsequent qRT-PCR detection.

### HuR stability assay

1 × 10^6^/ml MRC-5 cells were seeded in cell dishes. 2 ml serum-free medium containing 10 μg/ml cycloheximide was added into the cell samples. Cell proteins were extracted after cycloheximide treatment for different times. HuR and GAPDH protein levels were detected by Western blot.

### RNA-binding protein immunoprecipitation analysis

RIP assay was performed by using the RNA immunoprecipitation kit (GENESEED, China) according to the manufacturer’s instructions. The cell samples were collected and lysed by the lysis buffer containing protease inhibitors and RNase inhibitors. 100 μL supernatant was taken as input control, the negative control was normal rabbit anti-IgG antibody (Cell Signaling Technology, United States). The antibody was linked to the magnetic beads in advance to capture the antigen. Finally the RNA bound to the magnetic beads was eluted and purified for qRT-PCR analysis.

### Chromatin immunoprecipitation PCR analysis

CHIP- PCR assay was performed by using the SimpleChIP^®^ enzymatic chromatin IP kit (Cell Signaling Technology, United States) according to the manufacturer’s instructions. The cell samples were collected and incubated with formaldehyde for 10 min. 2.5 M glycine was added and mixed. After centrifugation, the precipitation was prepared for nuclear preparation and chromatin digestion. The chromatin was immunoprecipitated with anti-ATF3 or rabbit IgG antibodies and incubated overnight. 30 μl CHIP-grade protein G agarose beads was added and incubated for 2 h. NaCl and protease K were added and the enriched DNA-protein complex was de crosslinked to release DNA fragments. DNA was purified using a DNA centrifuge column and amplified by PCR. Primers for lncIAPF promoter were as follows: Forward-CTACCTTCAAGCCTTACTTCCTCCG, Reverse-GAATACAAGGCGCTATGCTAGGAAC.

### Statistical analysis

Data were expressed as the means ± standard deviation (SD) and analyzed using the GraphPad Prism statistic software program. Differences between groups were assessed by two-sided Student’s t-test. All experiments were repeated thrice. *p* < 0.05 was considered statistically significant.

## Results

### Danshensu methyl ester attenuated pulmonary fibrosis *In vivo* and *In vitro*

**FIGURE 2:**
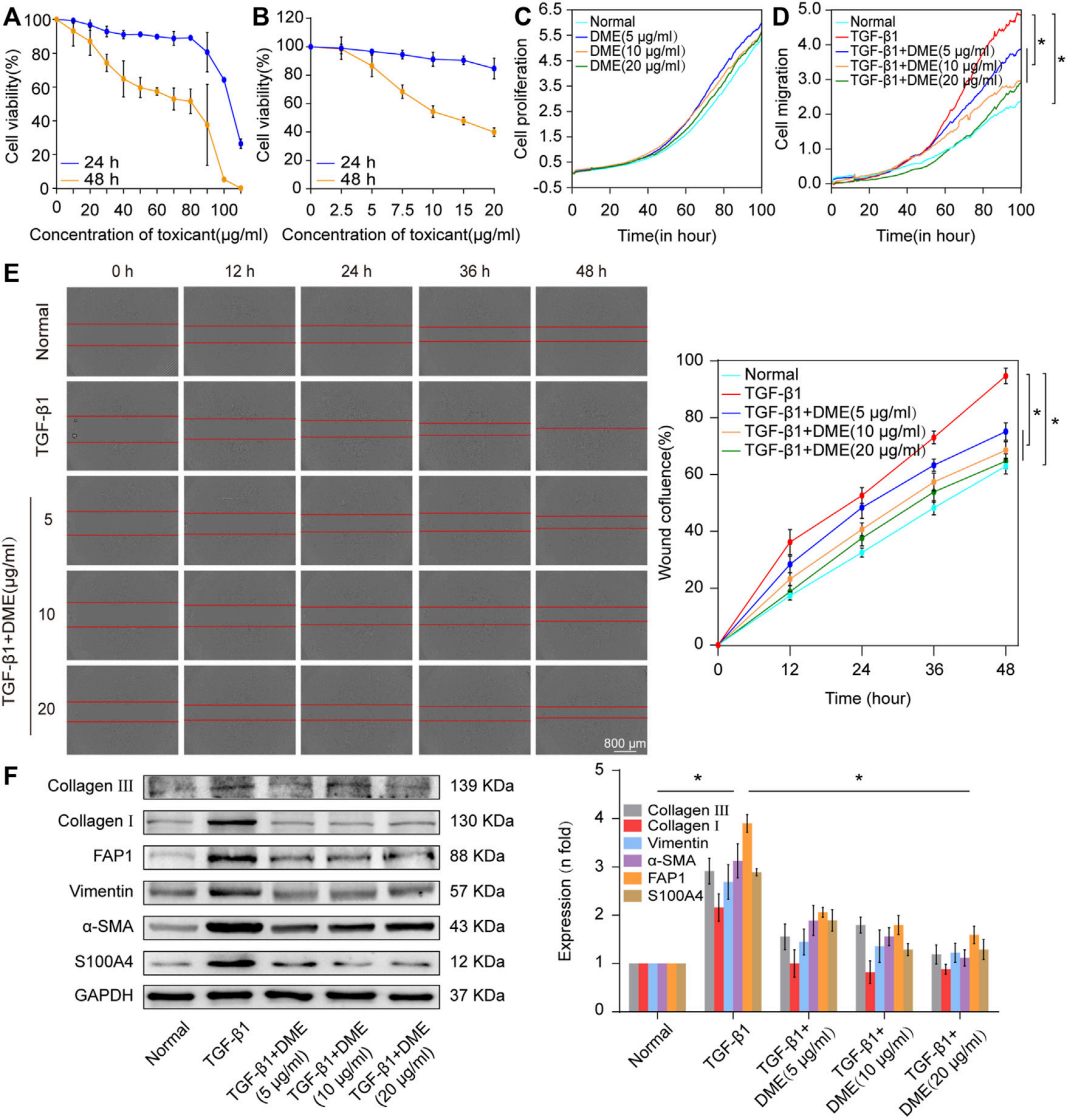
Concentration screening of DME and its anti-pulmonary fibrotic effect on MRC-5 cells. **(A)** CCK-8 was performed to test the drug toxicity of DME in normal MRC-5 cells. IC50 was approximately from 100 to 110 μg/ml under DME treated for 24 h and 80–90 μg/ml under DME treated for 48 h. **(B)** Inhibition of DME on TGF-β1-treated cells in a time- and dose-dependent manner. MRC-5 cells were initially cultured with 5 ng/ml of TGF-β1 for 72 h and then co-cultured with different concentrations of DME for 24 and 48 h respectively. **(C)** Real-time cell analysis identified that 5, 10, and 20 μg/ml of DME showed little toxicity against normal cell proliferation. **(D)** The curves of real-time cellular analysis revealed that 5, 10, and 20 μg/ml of DME significantly repressed the activated-fibroblast migration compared with those in the TGF-β1 treatment group **(E)** Analysis by using incuCyte S3 instrument confirmed that 5, 10, and 20 μg/ml of DME markedly inhibited the migration of cells treated with TGF-β1. **(F)** Western blot showed that DME decreased the expression level of S100A4, FAP1, vimentin, *a*-SMA, and collagen I and III. Each bar represents the mean ± SD; n = 6; *p < 0.05.

First, CCK-8 was used to evaluate the *in vitro* toxicity of DME. Human lung fibroblast MRC-5 cells were incubated in a medium containing different concentrations of DME for 24 and 48 h. Normal cell viability indicated that the IC50 of DME was approximately from 100 to 110 μg/ml at 24 h and 80–90 μg/ml at 48 h (Figure 2A). Then, 5 ng/ml of transforming growth factor β1 (TGF-β1) was used to establish a pulmonary fibrosis model in MRC-5 cells. MRC-5 cells were treated with TGF-β1 for 72 h, and then treated with different concentrations of DME (0, 2.5, 5, 7.5, 10, 15, 20 ug/mL) for 24 h and 48 h respectively. The results showed that DME inhibited TGF-β1-treated cells in a time- and concentration-dependent manner. Starting from the condition of 5 μg/ml DME-treated for 48 h, DME treatment showed a significant inhibitory effect (Figure 2B). The proliferation curve further confirmed that 5, 10, and 20 μg/ml of DME showed slight toxicity against normal cells (Figure 2C). Real-time cellular analysis (RTCA) experiment was performed to monitor the migration of TGF-β1-activated MRC-5 cells. The curves demonstrated that 5, 10, and 20 μg/ml of DME markedly inhibited the migration of cells treated with TGF-β1 (Figure 2D). The result of migration was further confirmed by scratch wound-healing assay by using an IncuCyte S3 instrument (Figure 2E). Western blot showed that DME decreased the expression level of differentiation-related proteins, including fibroblast activation protein 1 (FAP1) and S100 calcium-binding protein A4 (S100A4), and fibrotic markers, including vimentin, *α*-SMA, and collagen I and III (Figure 2F). Thus, 10 μg/ml of DME treated for 48 h was selected for further studies. These findings indicated that DME alleviated pulmonary fibrosis by inhibiting the differentiation of fibroblast into myofibroblast, myofibroblast proliferation and migration, and ECM deposition *in vitro*.

**FIGURE 3:**
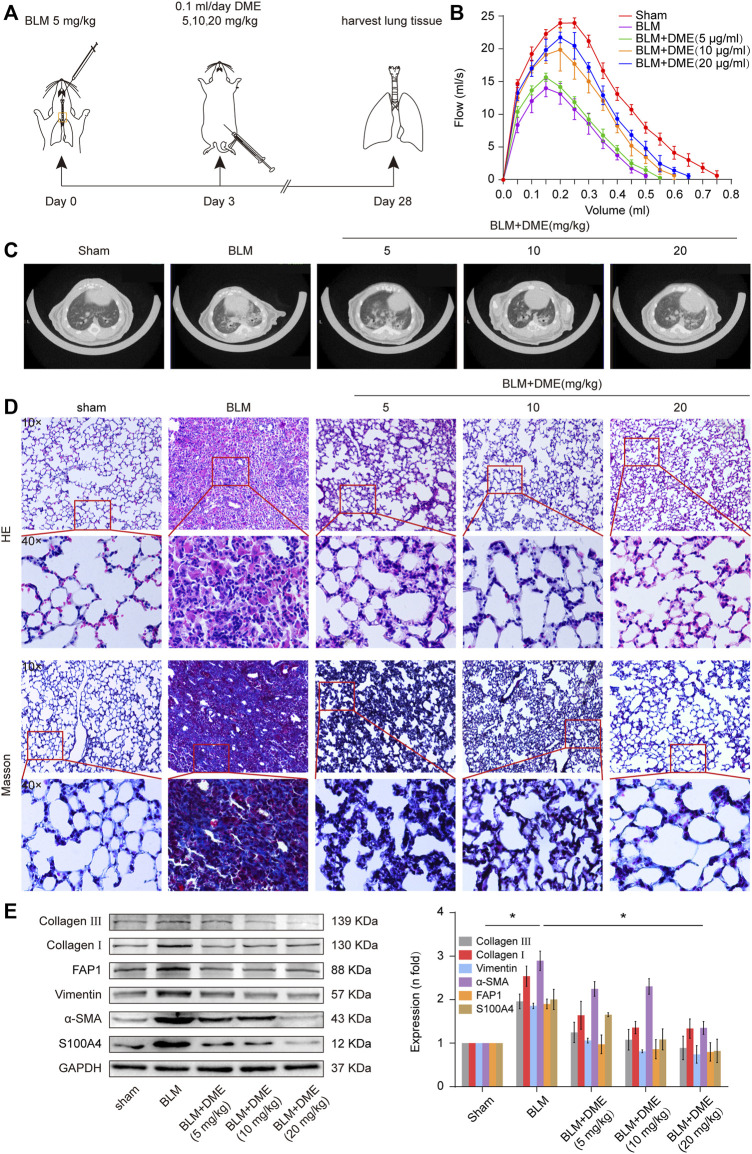
DME alleviated pulmonary fibrosis in BLM-treated mice. **(A)** Schematic illustration of DME injection to mice. **(B)** FVC results showed that DME enhanced the pulmonary function of mice compared with those in the BLM group. **(C)** MicroCT images exhibited that the BLM group had evident honeycomb-like changes and uneven patchy shadows compared with the sham group. The fibrotic symptoms were significantly improved in the DME group. **(D)** H&E and Masson staining unveiled that the alveolar structure was relatively complete; the alveolar septum became thinner, and collagen deposition was reduced in DME-treated mice compared with those of the BLM-treated group. **(E)** Western blot analysis showed that DME reduced the expression level of S100A4, FAP1, vimentin, *a*-SMA, and collagen I and III. Each bar represents the mean ± SD; *n* = 6; *p < 0.05.

Then, the anti-pulmonary fibrotic ability of DME was assessed in BLM-treated mice (Figure 3A). Lung-function assessment revealed that DME treatment obviously promoted the forced vital capacity (FVC) of mice (Figure 3B). The images of the MicroCT system for small animals depicted that the honeycomb lung and uneven patchy shadows were evident in the BLM-treated group. The fibrotic symptoms were remarkably alleviated under DME treatment (Figure 3C). H&E staining displayed that BLM-treated mice had evident collagen deposition, damaged alveolar structure, and mild inflammatory cell infiltration. After DME treatment, collagen deposition was reduced, and the alveolar structure was clear and complete. Masson staining demonstrated that the alveolar structure of the treatment group was more complete, and collagen deposition was reduced in DME-treated mice (Figure 3D). Western blot indicated that DME reduced the expression level of pulmonary fibrotic proteins, including vimentin, collagen, and *a*-SMA, and differentiation-related proteins, including FAP1 and S100A4 (Figure 3E). The abovementioned results indicated the anti-pulmonary fibrotic ability of DME *in vivo*.

### Danshensu methyl ester mitigated pulmonary fibrosis through downregulating lncIAPF–HuR complex to promote autophagy

**FIGURE 4:**
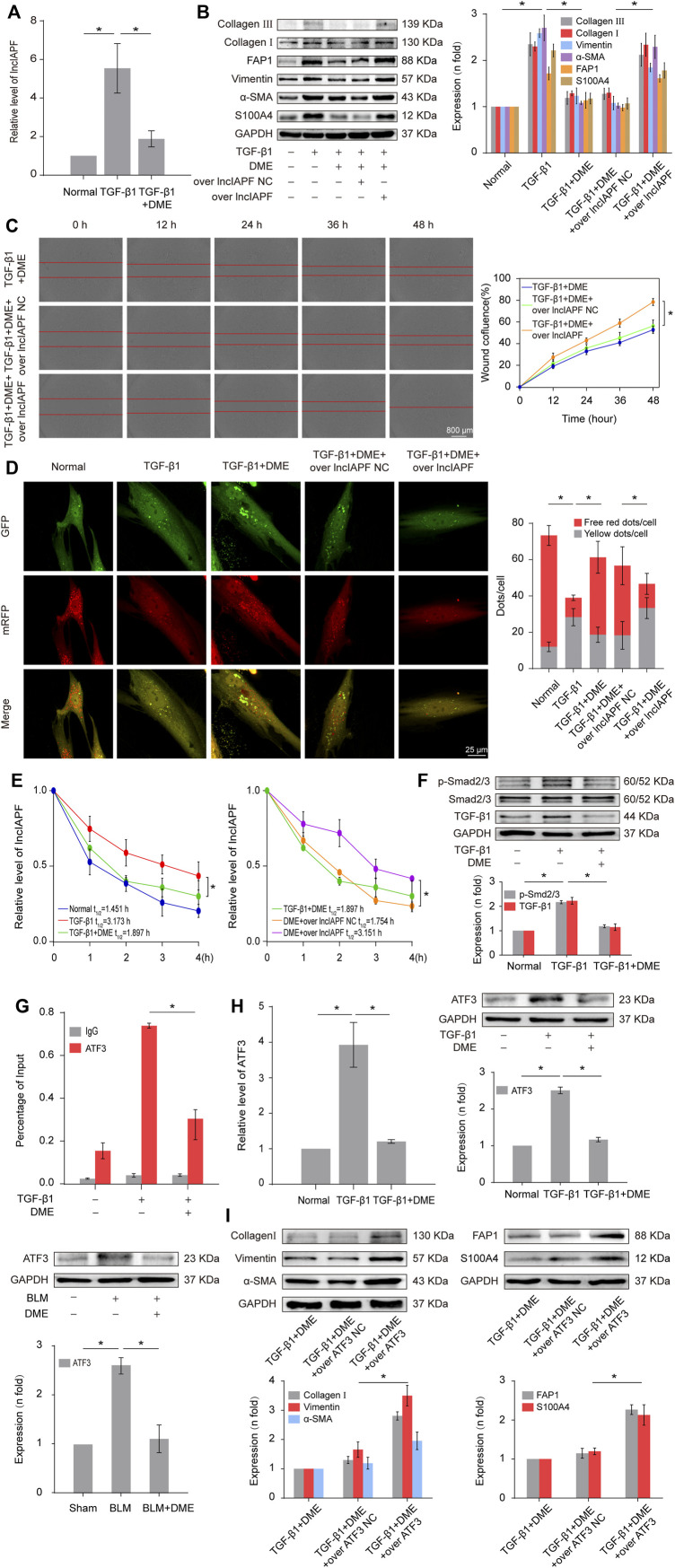
DME alleviated pulmonary fibrosis by weakening lncIAPF stability and transcription to promote autophagy. **(A)** qRT-PCR result showed that DME reduced the expression level of lncIAPF. **(B)** The rescue experiment of Western blot showed that DME reduced the expression of S100A4, FAP1, α-SMA, vimentin, and collagen I and III. lncIAPF overexpression increased the expression of S100A4, FAP1, *α*-SMA, vimentin, and collagen I and III and reversed the downward trend caused by DME. **(C)** The rescue experiment of scratch assay showed that lncIAPF overexpression reversed the downward trend of migration caused by DME. **(D)** Red fluorescence indicates autolysosomes in normal autophagy. Yellow fluorescence indicates autolysosomes in abnormal autophagy. The yellow dots increased in the TGF-β1 group compared with the normal group. The red dots increased in the DME group compared with the TGF-β1 group. **(E)** Actinomycin D experiment showed that lncIAPF gradually decreased with the prolongation of actinomycin D action. However, lncIAPF overexpression reversed this trend. **(F)** Western blot result showed that DME decreased the expression levels of TGF-β1 and p-Smad2/3. **(G)** CHIP-PCR showed that TGF-β1 enhanced the binding between the lncIAPF promoter region and ATF3. DME partially blocked the binding of the lncIAPF promoter region to ATF3. **(H)** qRT-PCR and Western blot results showed that DME decreased ATF3 expression at mRNA and protein levels. **(I)** The rescue experiment of Western blot showed that overexpression of ATF3 increased S100A4, FAP1, *α*-SMA, vimentin and collagen I and reversed the downward trend caused by DME. The concentration of DME used was 10 μg/ml. Over lncIAPF NC indicates a negative control. Each bar represents the mean ± SD; n = 6; *p < 0.05.

Autophagy is repressed in pulmonary fibrosis. lncIAPF–HuR complex can promote pulmonary fibrogenesis through inhibiting autophagy. Thus, we explored if lncIAPF–HuR could be the target for DME action. The results of qRT-PCR illustrated that DME reduced the expression level of lncIAPF (Figure 4A). An overexpressed plasmid of lncIAPF (over lncIAPF) and an empty vector without lncIAPF (lncIAPF negative control) were designed to transfect into MRC-5 cells. The rescue experiment of Western blot indicated that lncIAPF overexpression reversed the downward expression of S100A4, FAP1, *α*-SMA, vimentin, and collagen I and III caused by DME (Figure 4B). Moreover, the rescue experiment of scratch assay confirmed that lncIAPF overexpression reversed the therapeutic effect of DME (Figure 4C). The effect of DME on autophagy was tested by using the tandem dual-fluorescence HBAD-mcherry-EGFP-LC3 method. Red fluorescence indicates autolysosomes in normal autophagy. Yellow fluorescence indicates autolysosomes in abnormal autophagy, which suggests that autolysosomes can form, but can not be degraded and autophagy is partially blocked. The yellow dots increased in the TGF-β1 group compared with the normal group. The red dots increased in the DME group compared with the TGF-β1 group, which indicated that DME promoted autophagy. The rescue experiment further confirmed that lncIAPF overexpression reversed the enhanced effect of DME on autophagy (Figure 4D). Stable experiments demonstrated that DME weakened lncIAPF stability, and lncIAPF overexpression reversed the effect of DME on its stability (Figure 4E). The abovementioned findings indicated that DME attenuated pulmonary fibrosis *via* inhibiting lncIAPF to promote autophagy.

The regulatory mechanism of DME on the upstream signal pathway of lncIAPF was further explored. TGF-β1 signal transduction can be activated *via* the translocation of Smad2/3 in pulmonary fibrosis. Smad2/3 translocates from cytoplasm to nucleus *via* phosphorylation to realize TGF-β1 signal transduction. So we detected the expression of TGF-β1, Smad2/3 and p-Smad2/3. The result showed that DME inhibited TGF-β1 and p-Smad2/3, indicating DME inhibited TGF-β1 signal transduction directly (Figure 4F). Then, primers of the lncIAPF promoter were designed for CHIP-PCR to identify which transcription factor affected lncIAPF transcription under DME action. The data demonstrated that TGF-β1 enhanced the binding between the lncIAPF promoter region and activating transcription factor 3 (ATF3). The binding of the lncIAPF promoter region to ATF3 was partially blocked under DME action (Figure 4G). The results of qRT-PCR and Western blot further indicated that DME decreased ATF3 expression at mRNA and protein levels (Figure 4H). An overexpression plasmid ATF3 was designed and transfected into MRC-5 cells. The rescue experiment of Western blot indicated that ATF3 overexpression reversed the downward expression of S100A4, FAP1, *α* -SMA, vimentin, collagen I and collagen III caused by DME (Figure 4I). The abovementioned findings indicated that the inhibition of DME depended on ATF3 expression *via* TGF-β1 signal transduction.

**FIGURE 5:**
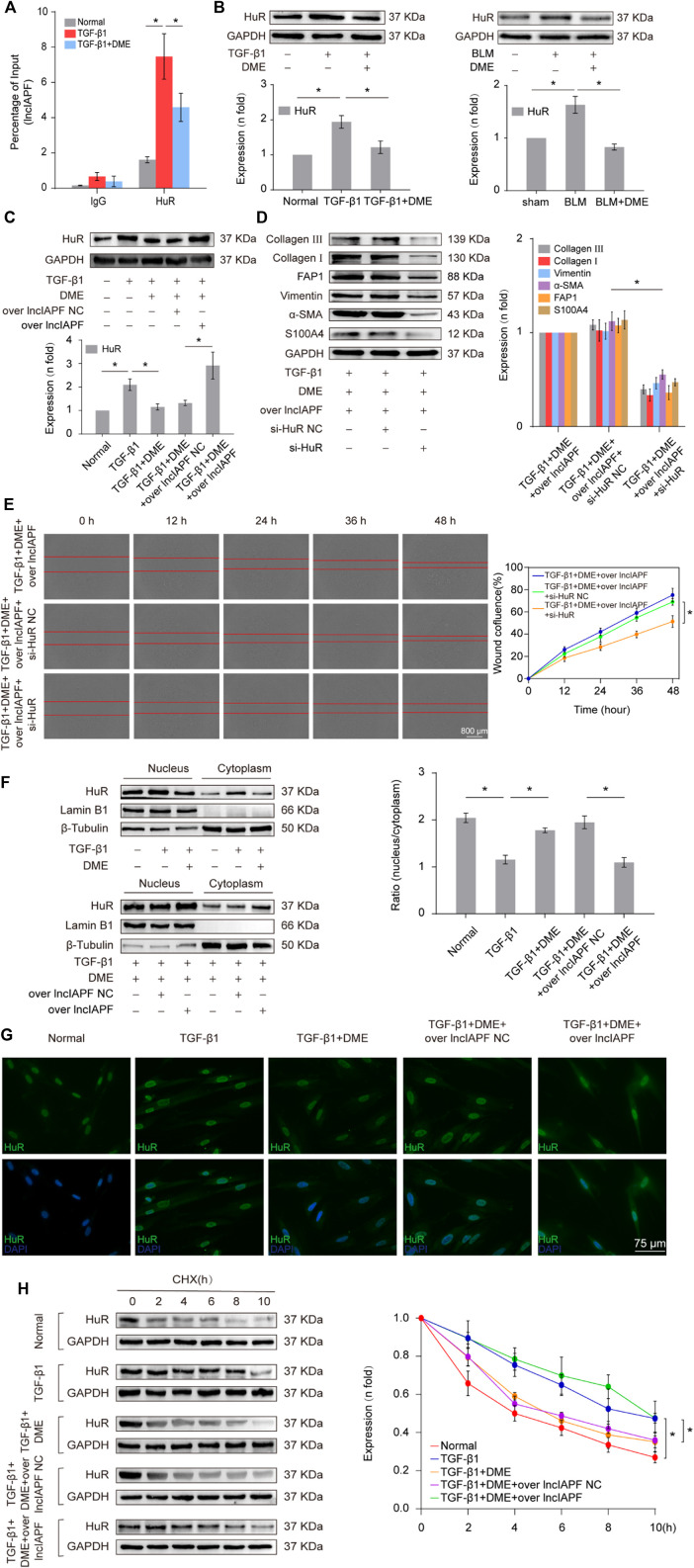
Regulatory mechanism of DME on lncIAPF–HuR. **(A)** The RIP experiment verified the binding relationship between lncIAPF and HuR and the effect of DME on their binding. **(B)** Western blot result showed that the expression of HuR increased in the model group and decreased in the treatment group. **(C)** The rescue experiment of Western blot showed that DME reduced HuR expression, and lncIAPF overexpression increased HuR expression and reversed the downward trend caused by DME. **(D)** The rescue experiment of Western blot showed that interference with HuR decreased the expression of S100A4, FAP1, α-SMA, vimentin, collagen I and III, and reversed the upward trend caused by lncIAPF overexpression. **(E)** The rescue experiment of scratch assay showed that HuR interinterference reversed the trend of accelerated migration caused by lncIAPF overexpression. **(F)** Nucleocytoplasmic separation experiment showed that DME blocked the nucleocytoplasmic translocation of HuR, but lncIAPF overexpression reversed the effect of DME. β-Tubulin was used as the cytoplasmic reference, and Lamin B1 was used as the nucleus. The results of nucleoplasmic separation were quantitatively analyzed by Image J software as follows: Normal: nucleus/plasm = 2.0, TGF-β1: nucleus/plasm = 1.3, TGF-β1+DME: nucleus/plasm = 1.8, TGF-β1+DME + overlncIAPF NC: nucleus/plasm = 1.9, TGF-β1+DME + overlncIAPF: nucleus/plasm = 1.1. **(G)** Immunofluorescence experiment showed that HuR was primarily localized in the nucleus of normal cells, and it transferred from the nucleus to the cytoplasm under the action of TGF-β1 or lncIAPF overexpression. DME blocked the nucleocytoplasmic translocation of HuR, but lncIAPF overexpression reversed the effect of DME. **(H)** Cycloheximide experiment verified the stability of the HuR protein. DME weakened HuR stability, but lncIAPF overexpression reversed this trend. The half-life of HuR in each group was presented as follows: normal: T1/2 = 3.07 h, TGF-β1: T1/2 = 10.17 h, DME: T1/2 = 3.92 h, DME + over lncIAPF NC: T1/2 = 4.76 h, DME + lncIAPF: T1/2 = 12.33 h. The concentration of DME used was 10 μg/ml. Each bar represents the mean ± SD; n = 6; *p < 0.05.

Then, an RNA immunoprecipitation (RIP) experiment was performed to test whether lncIAPF bound to HuR and investigate the effect of DME on their binding. The results confirmed that lncIAPF bound to HuR, and the binding amount increased under TGF-β1 action and decreased under DME treatment (Figure 5A). HuR expression was verified by Western blot experiment. HuR expression increased in the model group and decreased in the DME treatment group (Figure 5B). The rescue experiment further demonstrated that lncIAPF overexpression increased HuR expression and reversed the downward trend caused by DME (Figure 5C). Meanwhile, a small interfering RNA plasmid of HuR (si-HuR) was designed and transfected into MRC-5 cells after transfection of overexpressing lncIAPF. The rescue experiment of Western blot indicated that HuR interference reversed the high expression of S100A4, FAP1, *α*-SMA, vimentin, collagen type I and collagen type III induced by lncIAPF overexpression (Figure 5D). The rescue experiment of scratch assay showed that interference with HuR reversed the trend of accelerated migration caused by lncIAPF overexpression (Figure 5E). The data indicated that the effect of DME was dependent on lncIAPF- HuR complex. HuR, as an RNA-binding protein, can enhance the stability of lncIAPF and increase its expression. The regulatory mechanism of DME on HuR *via* lncIAPF was further explored. First, the location of HuR in the cell was detected by nucleocytoplasmic separation and immunofluorescence experiments. The results showed that HuR primarily existed in the nucleus of normal cells. Under the action of TGF-β1 or overexpressed lncIAPF, HuR was transferred from the nucleus to the cytoplasm. DME blocked HuR nucleocytoplasmic translocation, but lncIAPF overexpression reversed this effect (Figures 5F,G), indicating that DME blocked HuR nucleocytoplasmic translocation *via* lncIAPF. Cycloheximide experiment verified that DME weakened HuR stability depending on lncIAPF (Figure 5H). Collectively, these studies demonstrated that DME attenuated pulmonary fibrosis by inhibiting the lncIAPF–HuR-mediated autophagic signaling pathway.

### Danshensu methyl ester promoted autophagy by targeting the enhancer of zeste homolog 2, signal transducers and activators of transcription 1, and forkhead box K1

**FIGURE 6:**
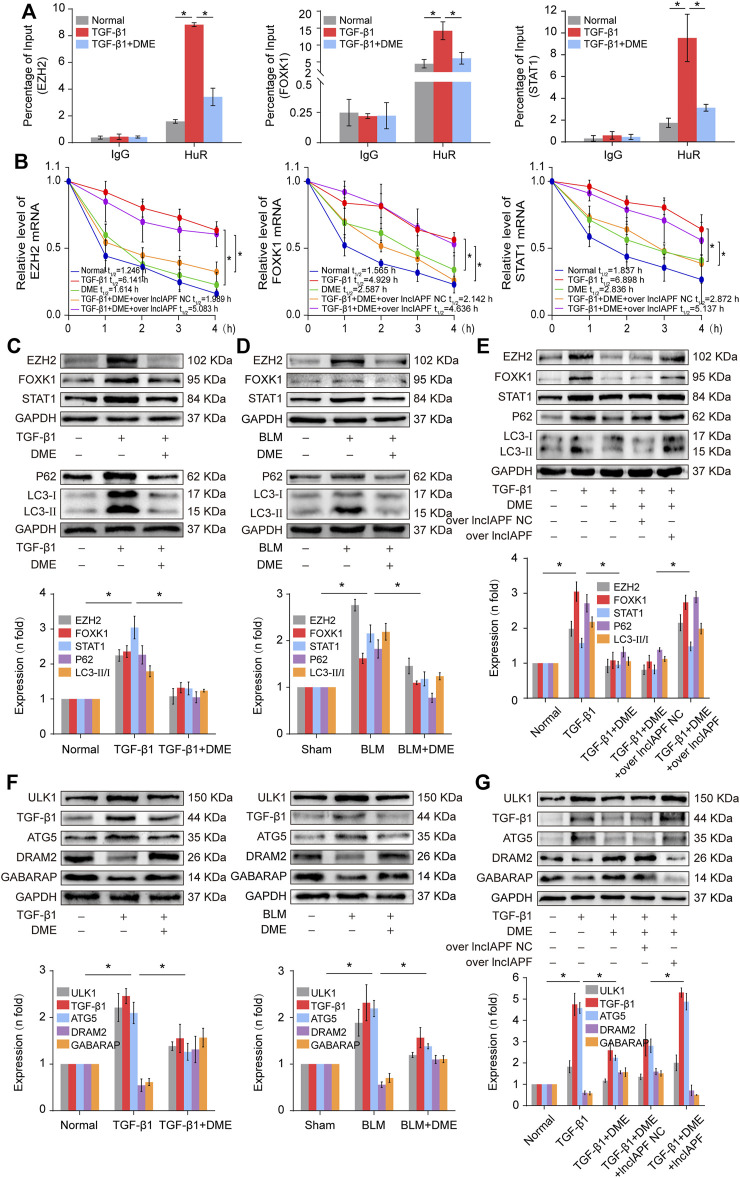
DME promoted autophagy through downregulating the target genes EZH2, STAT1, and FOXK1 depending on lncIAPF **(A)** RIP experiment showed that the mRNAs of EZH2, STAT1, and FOXK1 bound to HuR, and the binding amount decreased under DME treatment compared with TGF-β1 treatment **(B)** Half-life analysis revealed that DME treatment weakened the stability of EZH2, STAT1, and FOXK1 at the mRNA level, but lncIAPF overexpression reversed this trend. **(C)** Western blot result demonstrated that DME reduced the expression level of EZH2, STAT1, and FOXK1 in BLM-treated mice and TGF-β1-treated MRC-5 cells. **(D)** P62, LC3-II, and LC3-I expression levels in the DME group decreased compared with those in the TGF-β1/BLM-treated group. **(E)** The rescue experiment validated that lncIAPF overexpression reversed the effect of DME on target and autophagic genes. **(F)** Western blot identified that ATG5 and ULK1 decreased *in vivo* and *in vitro* in the DME group compared with those in the TGF-β1 group. In addition, GABARAP and DRAM2 levels increased *in vivo* and *in vitro* in the DME group compared with those in the TGF-β1 group. **(G)** The rescue experiment clarified that lncIAPF overexpression reversed the enhancement effect of DME treatment on autophagy. The concentration of DME used was 10 μg/ml. Each bar represents the mean ± SD; *n* = 6; *p < 0.05.

EZH2, STAT1, and FOXK1 are the target genes of HuR. Then, the effect of DME on autophagy through EZH2, STAT1, and FOXK1 was explored. The RIP experiment elucidated that the mRNAs of EZH2, STAT1, and FOXK1 bound to HuR, and the binding amount decreased under DME treatment compared with TGF-β1 treatment (Figure 6A). Half-life analysis revealed that DME treatment weakened the stability of EZH2, STAT1, and FOXK1 at the mRNA level, but lncIAPF overexpression reversed this trend, indicating that DME weakened the stability of EZH2, STAT1, and FOXK1 depending on lncIAPF (Figure 6B). Western blot detection showed that EZH2, STAT1, and FOXK1 decreased in the DME group compared with those in the TGF-β1/BLM group (Figure 6C). Autophagic marker proteins, such as P62, LC3I, and II, increased in the TGF-β1/BLM group and decreased in the DME group (Figure 6D). The rescue experiment showed that lncIAPF overexpression reversed this protein trend caused by DME, indicating that the enhancement effect of DME treatment on the target genes and autophagic flux was dependent on lncIAPF (Figure 6E).

Autophagosome accumulation was remarkably increased in pulmonary fibrosis (Figure 4D), indicating that autophagy was blocked in the later stage, and DME treatment promoted autophagy in pulmonary fibrosis. Thus, the effect of DME on autophagy was further explored. Autophagy-related gene 5 (ATG5) and unc-51-like kinase 1 (ULK1) play a role in the early stage of autophagy, and GABA type A receptor-associated protein (GABARAP) and DNA damage-regulated autophagy modulator 2 (DRAM2) contribute to the autophagosome–lysosome fusion in the later stage of autophagy. The Western blot result displayed that ATG5 and ULK1 decreased *in vivo* and *in vitro* in the DME group compared with those in the TGF-β1 group. Moreover, GABARAP and DRAM2 levels increased *in vivo* and *in vitro* in the DME group compared with those in the TGF-β1 group (Figure 6F). The rescue experiment verified that lncIAPF overexpression reversed this protein trend caused by DME, indicating that the enhancement effect of DME treatment on autophagy was dependent on lncIAPF (Figure 6G).

### Danshensu methyl ester alleviated pulmonary fibrosis *via* targeting lncIAPF–HuR-mediated autophagic signal pathway in mice

**FIGURE 7:**
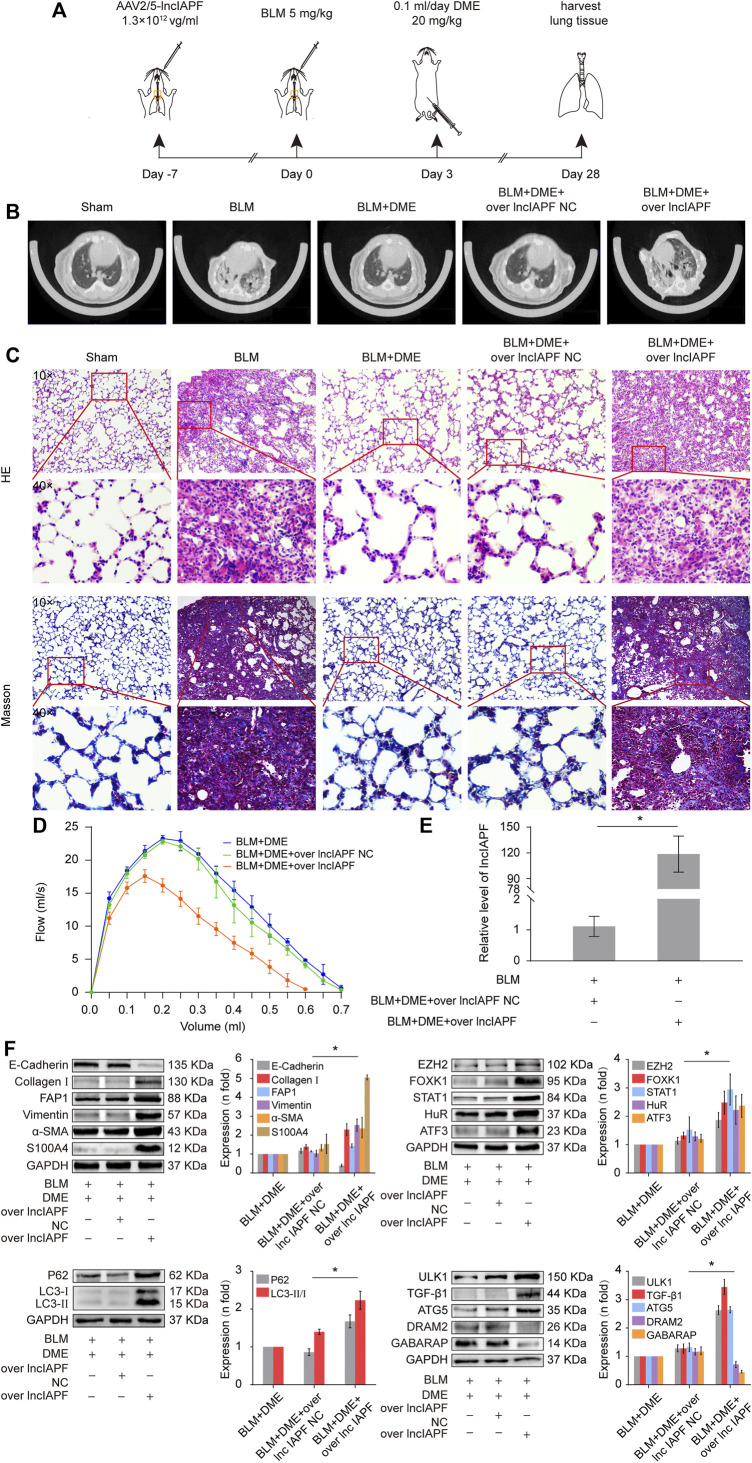
DME alleviated pulmonary fibrosis *via* downregulating the ATF3-lncIAPF–HuR–EZH2/STAT1/FOXK1 autophagic axis in mice **(A)** Schematic illustration of spraying overexpressed lncIAPF into mice **(B)** MicroCT images showed that the lungs of BLM-treated mice had evident honeycomb lung and uneven patchy shadows. DME treatment dramatically attenuated these fibrotic symptoms and lncIAPF overexpression reversed the effect of DME. **(C)** H&E and Masson staining showed that DME treatment lessened pulmonary fibrosis, but the alveolar walls in the lncIAPF overexpression group were thickened; collagen deposition increased, and the lung structure was changed. **(D)** FVC results showed that overexpression of lncIAPF worsened lung function and reversed the effect of DME on lung function. **(E)** qRT-PCR result showed that lncIAPF was highly expressed in the mouse model, indicating that adenovirus had been successfully constructed **(F)** In mice treated with overexpressed lncIAPF, the expression of fibrotic proteins (collagen, vimentin, and *a*-SMA) and autophagy-related proteins (EZH2, FOXK1, STAT1, P62, HuR, ATF3) increased, whereas that of E-cadherin, DRAM2, and GABARAP decreased compared with those in the BLM group. Each bar represents the mean ± SD; n = 6; *p < 0.05.

Rescue experiment was performed in mice to elucidate the action mechanism of DME targeting lncIAPF–HuR. The overexpressed lncIAPF was packaged into the adenovirus vector to spray into the mouse lung (Figure 7A). MicroCT images showed that the lungs of BLM-treated mice had evident honeycomb lung and uneven patchy shadows. DME treatment dramatically attenuated these fibrotic symptoms and lncIAPF overexpression reversed the effect of DME (Figure 7B). H&E and Masson staining presented the lung tissue structure and collagen deposition in mice. The results showed that DME treatment lessened pulmonary fibrosis, but the lncIAPF overexpression group had severe pulmonary fibrosis and more collagen deposition, which reversed the anti-pulmonary fibrosis of DME (Figure 7C). Lung function assessment revealed that lncIAPF overexpression worsened lung function, which reversed the effect of DME on lung function (Figure 7D). qRT-PCR data illustrated that lncIAPF overexpression increased lncIAPF expression and reversed the effect of DME on lncIAPF expression (Figure 7E). Western blot confirmed that lncIAPF overexpression increased the expression of fibrotic proteins (collagen, vimentin, and *a*-SMA), differentiation-related proteins (S100A4 and FAP1), and autophagy-related proteins (HuR, ATF3, EZH2, STAT1, FOXK1, P62, LC3I/II, and ULK1) and decreased DRAM2, GABARAP, and the epithelial marker protein E-cadherin, which reversed the effect of DME on these proteins (Figure 7F). The abovementioned finding indicated that DME alleviated pulmonary fibrosis in mice *via* downregulating the ATF3-lncIAPF–HuR–EZH2/STAT1/FOXK1 autophagic axis.

## Discussion

**FIGURE 8:**
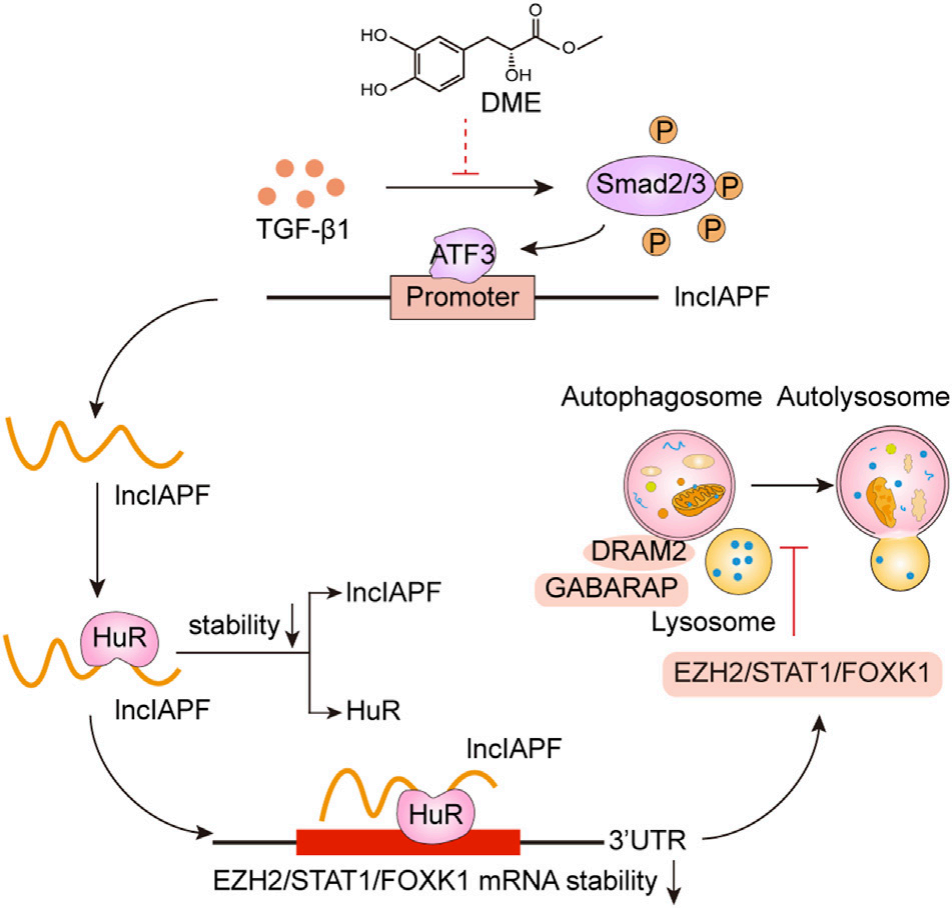
Therapeutic mechanism of DME treatment in pulmonary fibrosis.

Pulmonary fibrosis is characterized by fibroblast-to-myofibroblast differentiation, excessive proliferation and migration of myofibroblast, and accumulation of ECM components (Wu et al., 2020). Therefore, fibroblast-to-myofibroblast differentiation and excessive proliferation and migration of myofibroblast are the targets for anti-fibrotic drug development. For example, MBNL1 drives dynamic transition between fibroblasts and myofibroblasts, and tactical control of MBNL1 activity can alter fibrotic outcomes (Bugg et al., 2022). The methyl-CpG-binding domain 2 (MBD2) facilitates pulmonary fibrosis by orchestrating fibroblast-to-myofibroblast differentiation, and strategies aimed at silencing MBD2 can be potential therapeutic approaches for the prevention and treatment of pulmonary fibrosis (Wang et al., 2022). In addition, lncITPF accelerates fibroblast-to-myofibroblast differentiation to promote pulmonary fibrosis by targeting H3 and H4 histone acetylation in the ITGBL1 promoter depending on hnRNP-L (Song et al., 2019). Even, SARS-CoV-2 infection can drive fibroblast-to-myofibroblast transition leading to COVID-19 pulmonary fibrosis (Wang et al., 2021). Based on the results of atomic force microscopy, the mechanical stiffness of myofibroblast increases compared with normal fibroblast, which drives pulmonary fibrogenesis (Xu et al., 2022). In the present study, our results demonstrated that DME promoted autophagy to repress fibroblast-to-myofibroblast differentiation and proliferation and migration of myofibroblast *via* downregulating the lncIAPF–HuR-mediated autophagic axis (Figure 8). Mechanistically, DME inhibited ATF3 expression *via* TGF-β1 signal transduction leading to a decrease in lncIAPF transcription and stability. Moreover, DME blocked HuR nucleocytoplasmic translocation and promoted its degradation *via* downregulating lncIAPF, which markedly decreased the expression of HuR target genes such as negative autophagic regulators EZH2, STAT1, and FOXK1.

Autophagy is a normal cellular homeostatic process responsible for the lysosomal degradation of microorganisms, damaged organelles, and proteins that cannot be degraded by the ubiquitin–proteasome pathway (Klionsky et al., 2021; Ma et al., 2022). Abnormal autophagy contributes to the pathogenesis of human diseases targeting dysfunctional organelles, intracellular microbes, and pathogenic proteins (Levine and Kroemer., 2019). lncRNA-GBCDRlnc1 induces chemoresistance of gallbladder cancer cells by activating autophagy (Cai et al., 2019). The inhibition of lncRNA-Gm15834 attenuates autophagy-mediated myocardial hypertrophy *via* the miR-30b-3p/ULK1 axis in mice (Song et al., 2021). Under normal lung conditions, autophagy is critical for inhibiting spontaneous pulmonary inflammation and for the response of pulmonary stress. However, persistent and inefficient autophagy can promote lung injury (Racanelli et al., 2018). ATG4b-deficient mice displayed that autophagy disruption contributed to BLM-induced lung fibrosis (Cabrera et al., 2015). Annexin A2 is a specific BLM target to induce pulmonary fibrosis by impeding TFEB-mediated autophagic flux (Wang et al., 2018). Regarding lncRNA-related autophagy in pulmonary fibrosis, studies have reported that lncRNAs are involved in autophagy regulation. lncRNA-MEG3 restrained pulmonary fibrosis induced by NiO NPs *via* regulating autophagy mediated by the Hedgehog signaling pathway (Gao et al., 2022). lncIAPF promotes fibroblast-to-myofibroblast differentiation to accelerate pulmonary fibrosis through lncIAPF-mediated autophagic flux *in vivo* and *in vitro* in patients with idiopathic pulmonary fibrosis (IPF). The receiver operating characteristic curve (ROC) between lncIAPF and FVC shows that the sensitivity and specificity values are 87.5% and 75.0% in patients with IPF, respectively. The area under the ROC curve is 0.879 (Zhang J et al., 2022). In this study, we further proved that the lncIAPF–HuR complex can be a target related to autophagy for drug action. Autophagy is a continuous dynamic process and mainly contains 4 steps: formation of phagocytes, formation of autophage, formation of autophagosome and degradation of autophagosome. Our results showed that autophagosome accumulation was remarkably increased in pulmonary fibrosis, indicating that autophagy was blocked in the later stage, and DME treatment promoted autophagy *via* downregulating lncIAPF–HuR siganal pathway to mitigate pulmonary fibrosis.

HuR and its target genes EZH2, STAT1, and FOXK1 are negative autophagic regulators. m^6^A reader YTHDC1 interacts and cooperates with HuR in modulating autophagy by targeting SQSTM1 in diabetic skin (Liang et al., 2022). HuR usually serves as a RNA binding protein to regulate the stability and translation of messenger RNAs (Xiao et al., 2019). Our results indicated that DME weakened HuR stability to promote autophagy *via* inhibiting lncIAPF stability and transcription. The collected evidence indicates that overexpression or mutation of EZH2, STAT1, and FOXK1 is closely related to the occurrence and poor prognosis of multiple diseases; thus, they are attractive therapeutic targets for the treatment of diseases (Bowman et al., 2014; Kong et al., 2020; Patoli et al., 2020; Zhang J et al., 2022). Our mechanistic dissection revealed that DME inhibits EZH2, STAT1, and FOXK1 to block autophagy flux *via* weakening lncIAPF–HuR complex stability.

## Conclusion

In conclusion, the novel compound DME prepared by our group has remarkable anti-fibrotic effects *in vivo* and *in vitro*, and the lncIAPF–HuR can be the target for drug action. Our study provides valuable insights into the design of new drugs and presents candidate therapeutic targets for drug treatment.

## Data Availability

The original contributions presented in the study are included in the article/Supplementary Material, further inquiries can be directed to the corresponding authors.
