# Corrigendum: Danshensu methyl ester enhances autophagy to attenuate pulmonary fibrosis by targeting lncIAPF–HuR complex

**DOI:** 10.3389/fphar.2022.1086927

**Published:** 2022-12-02

**Authors:** Qi Zhu, Jing Wang, Yunxia Ji, Jianlin Luan, Dayong Yue, Weili Liu, Hongbo Li, Jinjin Zhang, Guiwu Qu, Changjun Lv, Xiaodong Song

**Affiliations:** ^1^ Department of Cellular and Genetic Medicine, School of Pharmaceutical Sciences, Binzhou Medical University, Yantai, China; ^2^ Department of Respiratory and Critical Care Medicine, Binzhou Medical University Hospital, Binzhou Medical University, Binzhou, China; ^3^ Medical Research Center, Binzhou Medical University, Yantai, China; ^4^ School of Gerontology, Binzhou Medical University, Yantai, China

**Keywords:** pulmonary fibrosis, danshensu, lncRNA, autophagy, HuR (ELAVL1)

In the published article, there was an error in Figure 5 as published. The quantitative grouping label of the protein bands in Figure 5D was incorrectly labeled as “TGF-β1+DME, TGF-β1+DME + si-HuR NC, TGF-β1+DME + si-HuR”. The corrected Figure 5 and its caption appear below.

**FIGURE 5:**
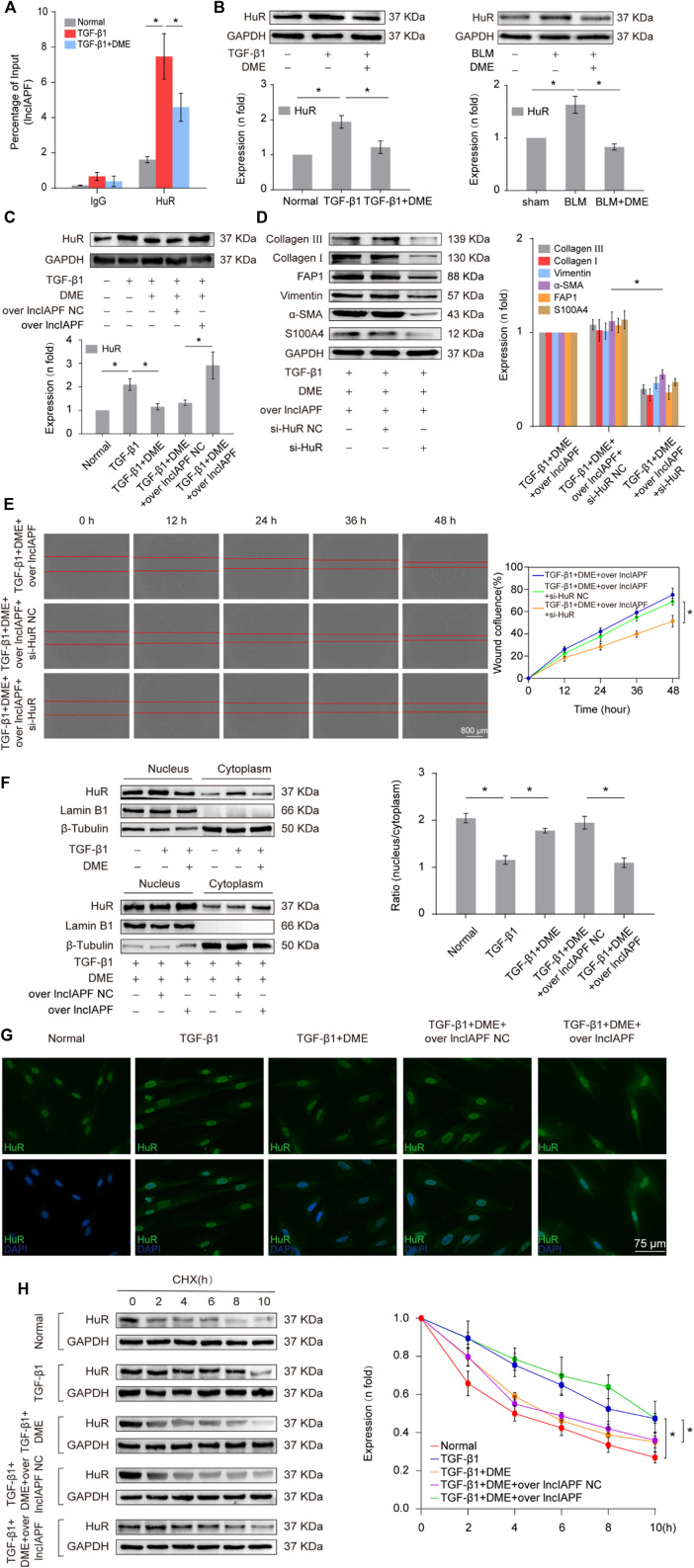
(Continued). Regulatory mechanism of DME on lncIAPF–HuR. **(A)** The RIP experiment verified the binding relationship between lncIAPF and HuR and the effect of DME on their binding. **(B)** Western blot result showed that the expression of HuR increased in the model group and decreased in the treatment group. **(C)** The rescue experiment of Western blot showed that DME reduced HuR expression, and lncIAPF overexpression increased HuR expression and reversed the downward trend caused by DME. **(D)** The rescue experiment of Western blot showed that interference with HuR decreased the expression of S100A4, FAP1, α-SMA, vimentin, collagen I and III, and reversed the upward trend caused by lncIAPF overexpression. **(E)** The rescue experiment of scratch assay showed that HuR interinterference reversed the trend of accelerated migration caused by lncIAPF overexpression. **(F)** Nucleocytoplasmic separation experiment showed that DME blocked the nucleocytoplasmic translocation of HuR, but lncIAPF overexpression reversed the effect of DME. β-Tubulin was used as the cytoplasmic reference, and Lamin B1 was used as the nucleus. The results of nucleoplasmic separation were quantitatively analyzed by Image J software as follows: Normal: nucleus/plasm = 2.0, TGF-β1: nucleus/plasm = 1.3, TGF-β1+DME: nucleus/plasm = 1.8, TGF-β1+DME + overlncIAPF NC: nucleus/plasm = 1.9, TGF-β1+DME + overlncIAPF: nucleus/plasm = 1.1. **(G)** Immunofluorescence experiment showed that HuR was primarily localized in the nucleus of normal cells, and it transferred from the nucleus to the cytoplasm under the action of TGF-β1 or lncIAPF overexpression. DME blocked the nucleocytoplasmic translocation of HuR, but lncIAPF overexpression reversed the effect of DME. **(H)** Cycloheximide experiment verified the stability of the HuR protein. DME weakened HuR stability, but lncIAPF overexpression reversed this trend. The half-life of HuR in each group was presented as follows: normal: T1/2 = 3.07 h, TGF-β1: T1/2 = 10.17 h, DME: T1/2 = 3.92 h, DME + over lncIAPF NC: T1/2 = 4.76 h, DME + lncIAPF: T1/2 = 12.33 h. The concentration of DME used was 10 μg/ml. Each bar represents the mean ± SD; n = 6; *p < 0.05.

The authors apologize for this error and state that this does not change the scientific conclusions of the article in any way. The original article has been updated.

